# Multi‐Parametric Biophysical Analysis for Evaluating Suitability of Energy‐Based Devices and Injectables

**DOI:** 10.1111/jocd.71224

**Published:** 2026-10-01

**Authors:** Jin Ho Kim, Nark‐Kyoung Rho, Jiyeoun Shin, Won Seok Choi

**Affiliations:** ^1^ V&co Cooperation Seoul Republic of Korea; ^2^ V‐Plastic Surgery Daegu Republic of Korea; ^3^ Leaders Dermatology Clinic Seoul Republic of Korea; ^4^ Hev Dermatology Clinic Seoul Republic of Korea

**Keywords:** energy –based device, skin biophysical property, skin rejuvenation

## Abstract

**Background:**

The paradigm of cosmetic dermatology has shifted heavily toward combination therapies involving energy‐based devices (EBDs) and injectable biostimulators. However, conventional efficacy assessments remain largely limited to subjective clinical grading or single‐parameter, uni‐dimensional approaches. Given the complex cross‐talk within the epidermal‐dermal microenvironment, a multi‐dimensional objective framework is required to achieve evidence‐based precision medicine.

**Aims:**

This study introduces a multi‐parametric biophysical stratification model to evaluate baseline patient suitability, reveal hidden antagonistic interferences (negative synergy), and establish an optimized treatment sequence for combined treatments.

**Methods:**

A retrospective cohort of 93 patients who underwent skin rejuvenation treatments was analyzed. Baseline (T0) biophysical profiles were measured via SKENA (V&Co., Seoul, Korea) with eight quantitative parameters: hydration, trans‐epidermal water loss (TEWL), dermal thickness via 10‐MHz ultrasound, erythema index, and elasticity parameters (R0, R2, R6/R5, R7). To eliminate multi‐collinearity and physiological cross‐talk, a Mahalanobis distance metric tensor utilizing the inverse covariance matrix was established. Modality‐specific suitability scores ($S_{m}$) and non‐linear combination synergy margins (Ω) were algebraically derived. A 12‐week longitudinal kinetic simulation was performed to compare concurrent and sequential treatment pathways.

**Results:**

Baseline stratification identified distinct phenotypic cohorts. EBD solo analysis separated patients into HIFU‐dominant (*N* = 51) and RF‐dominant (*N* = 42) cohorts, partitioned significantly by erythema (*p* = 0.0004) and viscoelastic creep (R6/R5, *p* = 0.0183). Scaffold analysis partitioned PN‐dominant (*N* = 48) and PLLA‐dominant (*N* = 45) cohorts via barrier integrity and dermal thinning (*p* = 0.0039). Incorporating an unclamped synergy model revealed robust supralinear synergy (Ω > 0) in barrier‐compromised phenotypes, where PN‐priming effectively harnessed EBD‐induced neocollagenesis. The model successfully rescued 14 patients from a “therapeutic desert” ($S_{m}$ < 45%) to a high‐suitability zone ($S_{\text{combination}}$> 75%). While the model identified potential antagonistic interferences (Ω ≤ 0) in approximately 50%–60% of cases involving PLLA when applied indiscriminately, these were localized to specific barrier‐disrupted phenotypes. Longitudinal kinetics demonstrated that a 4‐week sequential PN‐priming pathway completely bypassed the acute barrier crisis seen in concurrent pathways and achieved a significantly superior 12‐week net elasticity endpoint.

**Conclusions:**

Multi‐parametric biophysical stratification successfully translates complex interactions into actionable clinical guidelines. Recognizing negative synergy zones highlights the absolute clinical necessity of spatiotemporal sequential tracking rather than indiscriminate concurrent combinations, paving the way for data‐driven precision aesthetics.

## 1 Introduction

In recent years, skin rejuvenation interventions have largely emerged, driven by the rapid technological evolution of energy‐based devices (EBDs), such as microfocused ultrasound and radiofrequency, alongside advanced injectable biostimulators [1, 2]. To objectively evaluate the efficacy of these modalities, quantitative skin biophysical properties have been widely utilized in clinical research [1, 2]. However, conventional evaluation methodologies remain heavily confined to a uni‐dimensional approach, evaluating individual parameters—such as superficial hydration or elasticity—in isolation.

Such isolated assessments ignore the fundamental anatomical truth that the human skin exhibits a profound epidermal‐dermal structural complexity [3, 4]. The skin is a highly integrated system characterized by continuous, non‐linear biochemical and biomechanical interactions among various structural components within the epidermis and dermis. Reflecting this structural complexity, recent objective evidence confirms that distinct skin biophysical properties exhibit significant cross‐talk and correlations with one another [5]. Consequently, evaluating a single parameter without accounting for multi‐parametric covariance inevitably leads to confounding results and clinical misinterpretations.

For the successful adoption of evidence‐based precision medicine and the integration of future artificial intelligence frameworks within cosmetic dermatology, the utilization of comprehensive, quantitative values and their mathematical application is paramount. To transcend the inherent limitations of subjective clinical grading and uni‐dimensional evaluation approaches, a systemic model is required to map the complete baseline biophysical state of a patient. Because clinical skin biophysical variables are intrinsically interconnected through complex epidermal‐dermal cross‐talk, analyzing them in isolation introduces critical information redundancy and multi‐collinearity. To transcend these limitations, a systemic, multi‐parametric model is required to map the complete baseline state without statistical distortion.

## 2 Materials and Methods

### 2.1 Clinical Cohort and Multi‐Dimensional Biophysical Parameter Selection

This retrospective study utilized full baseline and longitudinal clinical datasets from a cohort of 93 patients who sought non‐surgical facial rejuvenation interventions. All patients wrote informed consent of acquisition and utilization of baseline biophysical properties. To minimize external environmental variance, all biophysical measurements were performed with SKENA (V&Co., Seoul, Korea; National Radio Research Agency Registration No. R‐R‐VNCO‐VSKINDX001). The operational validity, intra‐device repeatability, and concurrent correlation of this multi‐probe platform against globally established research standard instruments (Courage & Khazaka, Germany) were verified via an institutional‐approved pilot validation study, the full statistical details of which are provided in Supplementary 1. All measurements were done in a strictly climate‐controlled dark room (ambient temperature: 22°C ± 2°C, relative humidity: 45% ± 5%) after a mandatory 20‐min acclimation period.

For each patient i, an 8‐dimensional baseline state vector $X_{i}={\left[x_{i1},x_{i2},\cdots ,x_{i8}\right]}^{T}$ was constructed: Hydration ($x_{i1}$): Stratum corneum capacitance measured via hydration pin probe (μSiemens) [6]. TEWL ($x_{i2}$): Transepidermal water loss measured via TEWL probe (g/m^2^/h), reflecting epidermal barrier integrity [7, 8]. Dermal Thickness ($x_{i3}$): Absolute thickness of the dermis determined via 10‐MHz high‐resolution skin ultrasound [9]. Erythema ($x_{i4}$): Facial skin erythema index measured via colori probe (AU), indicating microvascular reactivity. R0 ($x_{i5}$): suction cup‐derived maximum skin extensibility under mechanical suction. R2 ($x_{i6}$): Gross skin elasticity (Ua/Uf ratio) derived via Cutometer. R6/R5 ($x_{i7}$): Viscoelastic creep ratio (Uv/Ue normalized by Ur/Ue), indicating structural tissue fluid mobility. R7 ($x_{i8}$): Net biological elasticity (Ur/Uf ratio), representing immediate elastic recovery [10, 11].

### 2.2 Mahalanobis Distance Transformation for Multi‐Collinearity Elimination

Prior to space transformation, Variance Inflation Factors (VIFs) were computed among raw biophysical parameters. Multiple indices (particularly between barrier integrity and viscoelastic creep) demonstrated significantly elevated VIF metrics (VIF > 5.0), confirming severe data collinearity. Standard Euclidean metrics are inherently heavily distorted by such data distribution. Consequently, a Mahalanobis distance metric tensor utilizing the inverse covariance matrix ($\Sigma^{-1}$) was established to mathematically cancel out overlapping variances and isolate true biophysical defect distances ($D_{M}$).

To eliminate physiological cross‐talk and multi‐collinearity among the eight biophysical variables, the baseline parameters were projected into a standardized Mahalanobis space. The raw vectors were first converted to standardized Z‐score vectors $z_{i}$:

$$z_{i}={\left[z_{i1}\;z_{i2}\cdots z_{i8}\right]}^{T},z_{\mathrm{ij}}=\frac{x_{\mathrm{ij}}-\mu_{j}}{\sigma_{j}}$$

where $\mu_{j}$ and $\sigma_{j}$ represent the cohort mean and standard deviation of parameter j, respectively. The inverse covariance matrix $\Sigma^{-1}$ of the cohort was computed and implemented as the foundational metric tensor:

$$D_{M}\left(z_{i}\right)=\sqrt{z_{i}^{T}\Sigma^{-1}z_{i}}$$

By utilizing $\Sigma^{-1}$ as the scaling kernel, overlapping variances between interconnected variables (e.g., the covariance between TEWL and viscoelastic creep) were algebraically canceled, isolating the true, unconfounded biophysical defect distance for each subject.

### 2.3 Derivation of Modality‐Specific Suitability Scores ($S_{m}$)

Mathematical models for individual modalities m (High‐Intensity Focused Ultrasound [HIFU], Radiofrequency [RF], Polynucleotide [PN], and Poly‐L‐Lactic Acid [PLLA]) were constructed by linking established literature‐derived clinical effect sizes (Cohen's d) with a dynamic clinical kinetic index ($\Lambda_{m,k}$) [12]. The raw therapeutic suitability score is derived as the net balance between the dynamic benefit function (${\text{Benefit}}_{m}$) and the downtime risk penalty function (${\text{Risk}}_{m}$):

$$\mathrm{Raw}\;{\text{score}}_{m}\left(z_{i}\right)={\text{Benefit}}_{m}\left(z_{i}\right)-{\text{Risk}}_{m}\left(z_{i}\right)$$

$${\text{Risk}}_{m}\left(z_{i}\right)=\sum_{k=1}^{8}z_{\mathrm{ki}}\cdot \Sigma^{-1}\cdot w_{m,k}$$

$$w_{m,k}=w_{m,k}^{\text{base}}\cdot \exp\left(\Lambda_{m,k}\cdot \max\left(0,z_{\mathrm{ki}}\right)\right)$$

For instance, the RF model incorporates severe penalty multipliers for baseline barrier disruption ($z_{2}$: TEWL, $\Lambda_{\mathrm{RF},2}$ = 1.35) and vascular hypersensitivity ($z_{4}$: Erytehma, $\Lambda_{\mathrm{RF},4}$ = 1.10), while the HIFU model bypasses the epidermal layer ($\Lambda_{\text{HIFU},2}$ = 0). Raw scores were mapped onto a logistic sigmoid function to output a normalized suitability percentage $S_{m}\in \left[0,100\%\right]$, set against the cohort median.

### 2.4 Unclamped Bidirectional Combination Synergy Margin Tensor (Ω)

The total suitability score for a combined multi‐modal prescription is governed by a non‐linear interaction margin tensor Ω:

$$S_{m_{1}+m_{2}}\left(z_{i}\right)=\max\left(S_{m_{1}}\left(z_{i}\right),S_{m_{2}}\left(z_{i}\right)\right)+\Omega_{\left(m1,m2\right)}\left(z_{i}\right)$$

Crucially, artificial mathematical constraints (max(0, ~)) were completely omitted from the synergy equation to fully open the phase space. The algebraic formulation allowed Ω to output both positive values (supralinear synergy bonus) and negative values (antagonistic interference/negative synergy). The tensor calculates the net sum between tissue‐matrix coupling benefits and cellular inflammatory overload costs based on the baseline state $z_{i}$:

$$\begin{array}{c} \Omega_{\left(m1,m2\right)}\left(z_{i}\right)=G_{\text{gain}}\left(m1,m2\right)\cdot \prod_{j\in \varepsilon }\max\left(0,\gamma_{j}\cdot z_{\mathrm{ji}}\right)\\ \;-R_{\text{risk}}\left(m1,m2\right)\cdot \sum_{k\in \upsilon }\max\left(0,\lambda_{k}\cdot z_{\mathrm{ki}}\right) \end{array}$$

where ε and υ define the specific biophysical parameter sets triggering mechanistic synergy or acute local hyper‐inflammation, respectively.

## 3 Results

### 3.1 Baseline Screening and Single Modality Phenotypic Bifurcation Patterns

The suitability screening of the 93‐patient cohort revealed distinct, non‐overlapping phenotypic boundaries. Within the single EBD treatment—HIFU or RF (Table 1), the model partitioned the cohort into an HIFU‐preferred group (*N* = 51) and an RF‐preferred group (*N* = 42). Independent‐samples *t*‐tests revealed highly significant differences in baseline facial erythema (*p* = 0.0004), viscoelastic creep (R6/R5, *p* = 0.0183), and net biological elasticity (R7, *p* = 0.0165), demonstrating that the model automatically filters out RF for vascular reactivity in sensitive skin type. In the single injectable—PN or PLLA (Table 2), the PN‐preferred group (*N* = 48) displayed severe baseline dehydration (*p* = 0.0242), barrier disruption (*p* = 0.0004), and high microvascular erythema (*p* < 0.0001). Conversely, the PLLA‐preferred group (*N* = 45) was defined by profound dermal atrophy (1.22 ± 0.11 mm, *p* = 0.0039), indicating that the model isolates PLLA suitability strictly to structurally depleted, non‐inflammatory skin.

**TABLE 1:** Comparative analysis of baseline skin biophysical properties in high HIFU‐suitability versus high RF‐suitability groups.

Parameters	HIFU preferred (*N* = 51)	RF preferred (*N* = 42)	*p*
Hydration (μS)	279.21 ± 73.42	287.07 ± 62.80	0.4625
TEWL (g/h/m^2^)	13.60 ± 5.36	11.87 ± 4.36	0.0765
Dermal thickness (mm)	1.27 ± 0.11	1.27 ± 0.11	0.6735
Erythema (A.U.)	18.93 ± 5.85	15.08 ± 5.61	**< 0.001**
R0 (mm)	2.56 ± 0.39	2.54 ± 0.34	0.6116
R2	0.87 ± 0.06	0.87 ± 0.05	0.6676
R6/R5	0.53 ± 0.22	0.44 ± 0.12	**0.0183**
R7	0.37 ± 0.1	0.33 ± 0.06	**0.0165**

*Note:* Values indicating statistically significant differences (*p* < 0.05) were formatted in bold.

**TABLE 2:** Comparative analysis of baseline skin biophysical properties in high PN‐suitability versus high PLLA‐suitability groups.

Parameters	PN preferred (*N* = 48)	PLLA preferred (*N* = 45)	*p*
Hydration (μS)	273.83 ± 60.00	292.28 ± 76.21	**0.0242**
TEWL (g/h/m^2^)	13.93 ± 5.53	11.63 ± 4.06	**< 0.001**
Dermal thickness (mm)	1.32 ± 0.06	1.22 ± 0.11	**0.0039**
Erythema (A.U.)	19.52 ± 6.16	14.70 ± 4.82	**< 0.001**
R0 (mm)	2.47 ± 0.35	2.63 ± 0.36	0.1492
R2	0.86 ± 0.06	0.88 ± 0.06	0.6770
R6/R5	0.54 ± 0.18	0.43 ± 0.18	0.2144
R7	0.32 ± 0.07	0.39 ± 0.10	0.5814

*Note:* Values indicating statistically significant differences (*p* < 0.05) were formatted in bold.

### 3.2 Quantification of Supralinear Synergy in EBD + PN Spaces

When EBDs were combined with PN, the non‐linear synergy margin Ω ignited selectively within barrier‐compromised, highly reactive baseline phenotypes (Figure 1). High‐synergy cohorts for HIFU+PN (*N* = 24, cutoff Ω > 6.07) and RF + PN (*N* = 23, cutoff Ω > 12.46) localized precisely in zones of elevated TEWL and microvascular erythema (Table 3 and Table 4). Furthermore, patients with significant baseline facial‐to‐forearm erythema gradients and low individual typological angles (ITA°, darker skin phenotypes) showed highly significant synergy responses, with a mean $\Omega_{\mathrm{RF}+\mathrm{PN}}$ = 8.88 points (*p* < 0.05).

**FIGURE 1:**
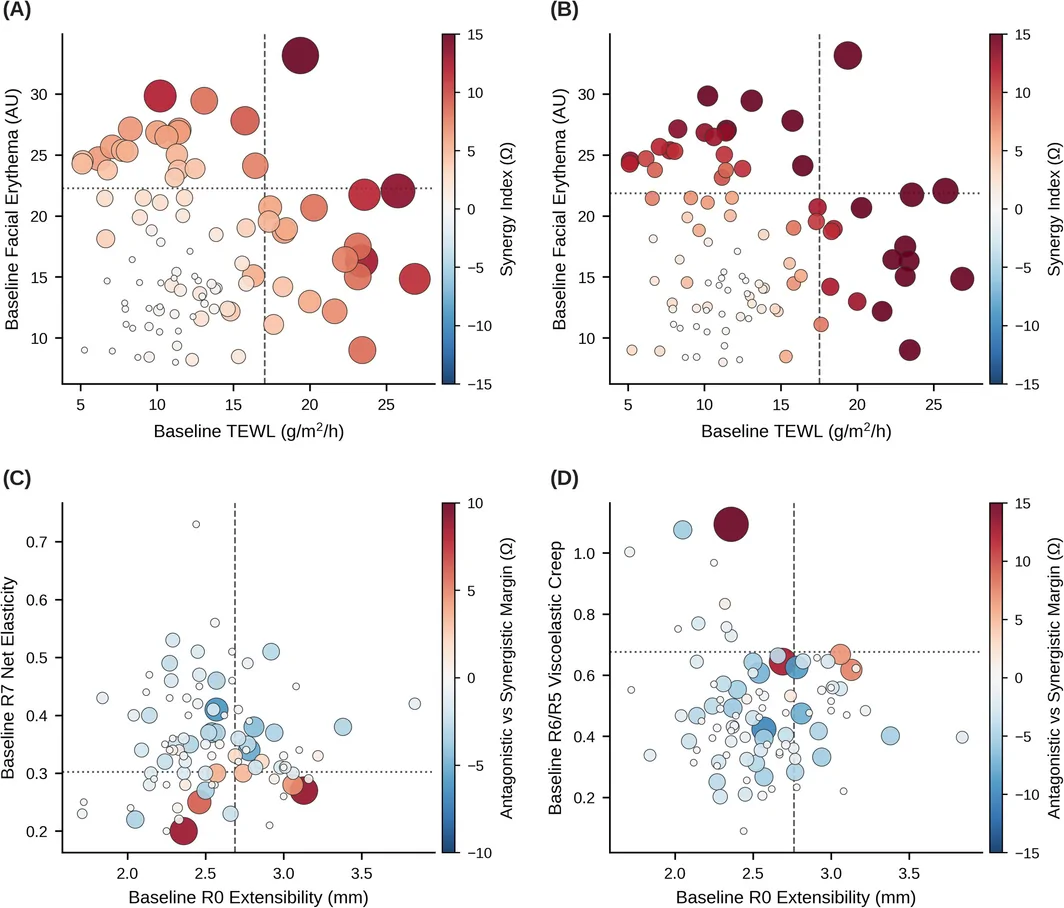
Bubble map of synergy index of (A) high‐intensity focused ultrasound (HIFU) combined with polynucleotide (PN) injection (B) monopolar radiofrequency (RF) combined with PN (C) HIFU combined with poly‐L‐lactic acid (PLLA) (D) RF combined with PLLA.

**TABLE 3:** Comparative analysis of baseline skin biophysical properties in High‐Synergy Score (Ω) for HIFU+PN and Low‐Synergy Score.

Parameters	High‐synergy (Ω>6.07, *N* = 24)	Low‐synergy (Ω≤6.07, *N* = 69)	*p*
Hydration (μS)	268.45 ± 65.12	286.91 ± 72.34	0.2745
TEWL (g/h/m^2^)	17.04 ± 6.42	11.35 ± 3.34	**< 0.001**
Dermal thickness (mm)	1.27 ± 0.10	1.27 ± 0.11	0.8974
Erythema (A.U.)	22.26 ± 6.19	15.43 ± 4.90	**< 0.001**
R0 (mm)	2.51 ± 0.35	2.56 ± 0.37	0.5421
R2	0.87 ± 0.05	0.87 ± 0.06	0.7245
R6/R5	0.51 ± 0.19	0.47 ± 0.18	0.3874
R7	0.34 ± 0.08	0.36 ± 0.09	0.4125

*Note:* Values indicating statistically significant differences (*p* < 0.05) were formatted in bold.

**TABLE 4:** Comparative analysis of baseline skin biophysical properties in High‐Synergy Score (Ω) for RF + PN and low‐synergy score.

Parameters	High‐synergy (Ω>12.46, *N* = 23)	Low‐synergy (Ω≤12.46, *N* = 70)	*p*
Hydration (μS)	265.14 ± 61.24	288.11 ± 71.94	0.1742
TEWL (g/h/m^2^)	17.51 ± 6.31	11.28 ± 3.27	**< 0.001**
Dermal thickness (mm)	1.27 ± 0.10	1.27 ± 0.11	0.8912
Erythema (A.U.)	21.86 ± 6.53	15.66 ± 5.02	**< 0.001**
R0 (mm)	2.50 ± 0.34	2.57 ± 0.37	0.4721
R2	0.87 ± 0.05	0.88 ± 0.06	0.6125
R6/R5	0.51 ± 0.19	0.47 ± 0.18	0.4194
R7	0.34 ± 0.08	0.36 ± 0.09	0.3412

*Note:* Values indicating statistically significant differences (*p* < 0.05) were formatted in bold.

### 3.3 Revelation of Negative Synergy and Antagonistic Interferences in PLLA Spaces

Removing the lower boundary clamp revealed that combining EBDs with PLLA induced profound negative synergy across the majority of the phase space. In HIFU combined with PLLA, the mean cohort Ω was −0.5488 ± 2.3226 points, with 53.8% of the cohort (50 patients) sinking into negative synergy (minimum extreme: −5.98 points). Significant differences were confirmed across dermal thickness, erythema, R0, R2, and R7 (*p* < 0.05, Table 5). In RF combined with PLLA, the mean cohort Ω plunged to −1.2845 ± 3.8487 points, with 60.2% of the cohort (56 patients) trapped in an antagonistic zone that actively degraded single‐treatment suitability (minimum extreme: −9.97 points, Table 6). The 56 patients experiencing negative antagonism in the RF + PLLA space presented a baseline mean TEWL of 14.20 g/m^2^/h and erythema of 17.50 A.U., demonstrating that baseline barrier disruption maps directly to negative synergy when exposed to simultaneous thermal and particulate stress.

**TABLE 5:** Comparative analysis of baseline skin biophysical properties in Positive Synergy Score (Ω) for HIFU+PLLA and Negative Synergy Score.

Parameters	Positive‐synergy (Ω>0, *N* = 43)	Negative‐synergy (Ω≤0, *N* = 50)	*p*
Hydration (μS)	288.42 ± 74.22	277.24 ± 65.41	0.4412
TEWL (g/h/m^2^)	12.14 ± 4.21	13.41 ± 5.14	0.1984
Dermal thickness (mm)	1.27 ± 0.11	1.38 ± 0.10	**< 0.001**
Erythema (A.U.)	16.90 ± 5.80	17.30 ± 6.10	**0.0401**
R0 (mm)	2.53 ± 0.35	2.56 ± 0.39	**0.0397**
R2	0.87 ± 0.06	0.87 ± 0.05	0.095
R6/R5	0.46 ± 0.16	0.50 ± 0.20	0.2874
R7	0.34 ± 0.08	0.36 ± 0.09	**< 0.001**

*Note:* Values indicating statistically significant differences (*p* < 0.05) were formatted in bold.

**TABLE 6:** Comparative analysis of baseline skin biophysical properties in Positive Synergy Score (Ω) for RF + PLLA and Negative Synergy Score.

Parameters	Positive‐synergy (Ω>0, *N* = 37)	Negative‐synergy (Ω≤0, *N* = 56)	*p*
Hydration (μS)	290.14 ± 76.12	278.14 ± 64.91	0.4125
TEWL (g/h/m^2^)	11.85 ± 3.99	14.20 ± 5.34	0.0625
Dermal thickness (mm)	1.24 ± 0.10	1.29 ± 0.09	**0.0039**
Erythema (A.U.)	16.50 ± 5.90	17.50 ± 6.00	**0.0256**
R0 (mm)	2.51 ± 0.34	2.56 ± 0.39	0.5412
R2	0.87 ± 0.06	0.87 ± 0.05	0.7412
R6/R5	0.45 ± 0.15	0.50 ± 0.19	**0.0082**
R7	0.33 ± 0.07	0.37 ± 0.09	**< 0.001**

*Note:* Values indicating statistically significant differences (*p* < 0.05) were formatted in bold.

### 3.4 Rescue Rate Within the “Therapeutic Desert”

The state‐space model successfully isolated 14 severe patients marooned in a “therapeutic desert” defined by baseline single suitability scores below 45% (mean baseline $S_{\text{HIFU}}$ = 20.681%, $S_{\mathrm{RF}}$ = 13.703%). By projecting these non‐responders onto the multi‐modal interaction plane, the combination formula pulled 100% of these high‐risk patients out of the desert, upgrading them into a secure clinical response zone ($S_{\text{combination}}$> 75%).

### 3.5 Spatiotemporal Trajectory Optimization via Longitudinal Kinetics Simulation

Longitudinal 12‐week kinetic modeling of the 14 rescued patients demonstrated a profound divergence based on treatment sequencing. For Concurrent Path (T0: RF + PN simultaneously): Induced an immediate, severe epidermal crisis at week 2, with TEWL spiking to 25.58 ± 4.2 g/m^2^/h, accompanied by prolonged erythema. The final week 12 biological recovery was truncated at R7 = 0.49 ± 0.08. For Sequential Path (RF intervention 4 weeks after PN injection): Bypassed the week 2 barrier crisis entirely, maintaining a stable TEWL of 12.5 ± 2.0 g/m^2^/h at week 2 and 11.2 ± 1.5 g/m^2^/h at week 4. Following RF intervention at week 4, only a mild, transient TEWL fluctuation (15.8 ± 2.2) occurred at week 8. Crucially, the sequential pathway achieved a significantly superior mechanical endpoint at week 12, reaching R7 = 0.57 ± 0.06 (*p* < 0.01). (Figure 2).

**FIGURE 2:**
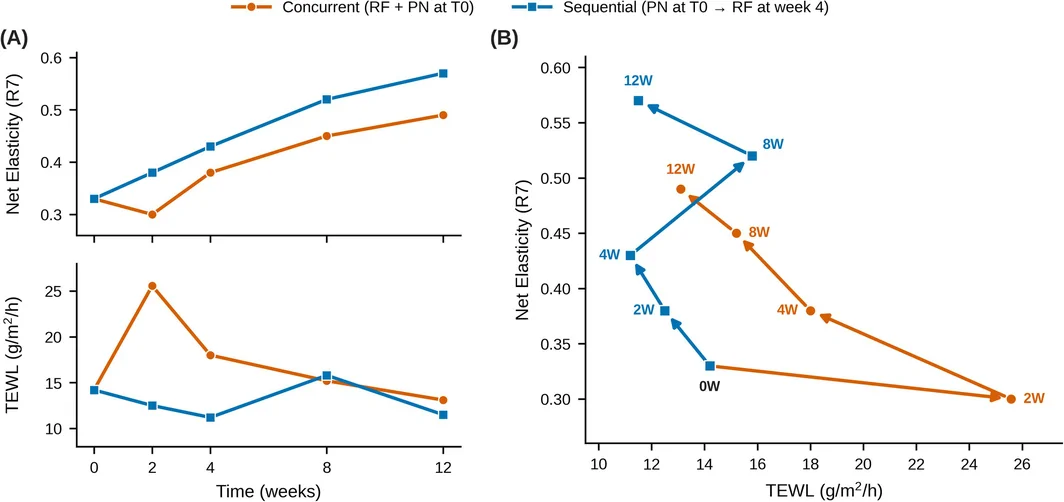
Longitudinal 12‐week kinetic modeling of the 14 rescued patients demonstrated a profound divergence based on treatment sequencing for concurrent Path (T0: RF + PN simultaneously) and sequential Path (RF intervention 4 weeks after PN injection). Panel (A) now describes the time course of net elasticity (R7) and TEWL over 12 weeks, and panel (B) describes the TEWL–R7 trajectory of the concurrent and sequential paths.

## 4 Discussion

The core advancement of the multi‐parametric state‐space model lies in its ability to mathematically resolve the multi‐collinearity inherent to skin biophysical measurements. In clinical cosmetic dermatology, individual skin parameters are frequently treated as independent metrics. However, as demonstrated by [1], skin properties are highly correlated due to the structural complexity of the epidermal‐dermal matrix [1]. For example, a severe defect in stratum corneum hydration ($x_{1}$) or an increase in transepidermal water loss ($x_{2}$) alters the interstitial fluid volume within the underlying dermis, directly distorting suction cup‐derived mechanical metrics such as viscoelastic creep (R6/R5) and net elasticity (R7) [13, 14].

By implementing a Mahalanobis metric tensor through the inverse covariance matrix ($\Sigma^{-1}$), our framework mathematically neutralizes this physiological cross‐talk. It subtracts overlapping variance structures, isolating the unconfounded vector distance ($D_{M}$) of a patient's true biophysical deficit. The resulting suitability profiles ($S_{m}$) and synergy metrics (Ω) are derived without arbitrary scaling, utilizing literature‐verified effect sizes to transform raw data into an objective clinical decision support system.

The distinct patterns revealed in Table 1 and Table 2 mirror the fundamental histopathological actions and downtime kinetics of the each treatment. In the monotherapy of EBD, the model directed patients with high baseline erythema (18.932 ± 5.850 vs. 15.075 ± 5.613, *p* = 0.0004) and high viscoelastic creep away from RF and toward HIFU. Radiofrequency applications rely on the delivery of electrical current across the electrical impedance of the epidermis, which inevitably generates localized thermal stress at the dermo‐epidermal junction [15, 16]. In a barrier‐compromised skin phenotype, this thermal load acts as a kinetic accelerator of inflammation, breaking down epidermal tight junctions and inducing microvascular ectasia. The model's risk function detects this susceptibility by running an exponential penalty calculation expΛ·z, automatically suppressing the RF suitability score. Conversely, HIFU delivers focused acoustic energy directly into the deep dermis or superficial muscular aponeurotic system (SMAS), bypassing the epidermis entirely ($\Lambda_{\text{HIFU},2}$ = 0), thereby rendering it a safer, superior selection for sensitive skin [17, 18].

A similar gatekeeping logic is apparent in the isolated scaffold screening. The model isolated PLLA suitability strictly to non‐inflammatory patients with profound dermal thinning (1.216 ± 0.114 mm), *p* = 0.0039, while assigning barrier‐disrupted, high‐erythema patients to PN. Mechanistically, PLLA microspheres act via a controlled subclinical foreign‐body reaction, stimulating host macrophages to release fibroblastic growth factors for delayed neocollagenesis [19, 20]. If PLLA is introduced into an actively inflamed, vascularly unstable microenvironment, the elevated baseline cytokine cascade shifts macrophage polarization toward an unmitigated M1 pro‐inflammatory phenotype, severely compounding the risk of nodule formation and sterile granulomas. Conversely, PN acts via purinergic A2A receptor pathways to exert non‐thermal, anti‐inflammatory matrix restoration, safely dampening baseline inflammation while accelerating early viscoelastic recovery, as corroborated by established literature [21, 22].

The increase of supralinear synergy in the EBD + PN spaces (Table 3 and Table 4) highlights a clinical intersection. High‐synergy cohorts localized in patients with severe baseline barrier damage (TEWL > 17 g/m^2^/h) and high microvascular erythema. Thermal stress from EBDs normally upregulates matrix metalloproteinases (MMPs), which degrade newly synthesized collagen fibers. However, when PN is combined with an EBD in a compromised microenvironment, its purinergic signaling pathways actively downregulate pro‐inflammatory cytokines (IL‐1β, TNF‐α) and upregulate IL‐10. This “gatekeeper risk offset” protects the newly stimulated fibroblastic zone, allowing the thermal neocollagenesis vectors of the EBD to achieve maximum mechanical expression without destructive inflammatory feedback loops. This phenomenon is further verified by the significant synergy spikes observed in highly reactive photoaged and darker skin phenotypes (*p* < 0.05), where neurovascular stability is paramount.

Conversely, the emergence of widespread negative synergy (Ω≤0) in the PLLA combination spaces (Table 5 and Table 6)—affecting 53.8% of HIFU+PLLA and 60.2% of RF + PLLA patients—represents a critical clinical warning. Although EBD thermal restructuring and PLLA‐mediated neocollagenesis represent orthogonal biochemical pathways that should theoretically double the therapeutic yield, simultaneous application into a barrier‐disrupted skin state (TEWL = 14.20, Erythema = 20.08) pushes the local homeostatic threshold past its breakdown point.

The immediate thermal stress of the EBD and the persistent particulate foreign‐body stress of the PLLA microspheres combine to lock local macrophages into a chronic M1 destructive phase. This prolonged pro‐inflammatory state drives the fusion of foreign‐body giant cells, leading to a pathological cellular microenvironment that degrades local elastic fibers and destabilizes tight junctions, causing a chronic rise in TEWL and microvascular inflammation [23, 24]. The suitability score drops drastically, losing up to −9.97 points. Positive synergy was preserved only in a highly restricted subset of patients presenting extreme baseline skin laxity (R0 > 3.1 mm, *p* = 0.0397) and zero microvascular reactivity, where the absolute requirement for structural volume overrides the cellular risk penalty.

The kinetic simulation of the 14 “therapeutic desert” non‐responders provides definitive proof of the necessity of spatiotemporal sequencing. Concurrent application of RF and PLLA or PN at T0 forces a severe biological collision, resulting in a week 2 TEWL crisis (25.58 ± 4.2) and a compromised final elastic recovery (R7 = 0.49 ± 0.08). This occurs because the tissue's metabolic capacity is overwhelmed by simultaneous thermal and mechanical trauma. In contrast, the Sequential path utilizes a 4‐week PN‐priming phase to systematically reshape the patient's biophysical coordinates. The purinergic anti‐inflammatory and barrier‐stabilizing kinetics of PN safely pull the patient out of the pro‐inflammatory zone, bringing TEWL and erythema parameters to baseline‐normal levels ($z_{2},z_{4}\leq 0$) by week 4. When the high‐energy EBD or PLLA is subsequently introduced at week 4, the exponential penalty functions are completely defused. The skin microenvironment, now stabilized and structurally primed, absorbs the thermal neocollagenesis vectors with minimal inflammatory resistance. This sequence achieves a vastly superior mechanical endpoint at week 12 (R7 = 0.57 ± 0.06, *p* < 0.01). These findings demonstrate that optimal combination therapy in cosmetic dermatology is not an act of simultaneous addition but rather a carefully calculated spatiotemporal trajectory that sequences treatments to exploit biological windows of safety and synergy.

Despite the robust mathematical framework and statistically significant outcomes established in this study, several limitations must be acknowledged. First, the retrospective nature and single‐center design involving a cohort of 93 patients restrict the immediate generalizability of the findings across heterogeneous ethnic or demographic groups. Prospective, multi‐center trials are required to rigorously validate these boundaries. Second, while the pathophysiological underpinnings of negative synergy—such as the M1 macrophage polarization cascade and tight junction degradation—were comprehensively detailed, these cellular shifts were inferred from non‐invasive surface parameter changes rather than confirmed through direct, invasive histopathological or immunohistochemical analysis.

To overcome these boundaries without burdening the clinical workflow with prospective punch biopsies, future research directions will focus on non‐invasive algorithmic scaling. Immediate efforts include training explainable artificial intelligence (XAI) architectures, such as SHAP‐augmented gradient boosting machines, to automatically map baseline variables onto clinical risk boundaries. Additionally, expanding the metric tensor by fusing multi‐modal digital assets, including optical cross‐polarized facial photography and UV fluorescence imaging, with current biophysical variables will refine predictive fidelity.

## 5 Conclusion

This study successfully demonstrates that a multi‐parametric biophysical stratification model can effectively translate complex, non‐linear skin interactions into a reliable clinical navigation framework. By eliminating variable multi‐collinearity through a Mahalanobis metric tensor, the model maps precise solo‐modality boundaries and exposes significant zones of negative synergy when EBDs and biostimulators are combined indiscriminately. The longitudinal kinetic simulation establishes that a 4‐week sequential priming strategy safely bypasses acute epidermal crises and maximizes biomechanical recovery compared to concurrent combinations. This algorithmic framework provides a foundational stepping stone toward evidence‐based, data‐driven precision aesthetics, enabling clinicians to replace empirical intuition with quantitative predictability.

## Author Contributions

Dr. Jin Ho Kim contributed to all investigation processes, data analysis, and writing the draft. Dr. Won Seok Choi contributed to all investigation processes, Conceptualization. Dr. Nark‐Kyoung Rho and Dr. Jiyeon Shin conceptualized and executed the clinical validation study provided in Supplementary 1.

## Funding

The authors have nothing to report.

## Consent

All participants provided written informed consent.

## Conflicts of Interest

First author is an employee of V&Co. The corresponding author is the Chief Executive Officer (CEO) of V&Co. Co‐authors have served as clinical consultants and Key Opinion Leaders (KOLs) for V&Co. The diagnostic tool (SKENA) utilized for clinical data harvesting in this retrospective analysis is manufactured by V&Co. The authors explicitly declare that this study was executed with strict scientific objectivity; the primary objective remains the academic introduction of the multi‐parametric suitability matrix and spatiotemporal optimization rules, completely independent of any commercial product promotion.

## Supporting information


**Supplementary 1** Technical Validation, Repeatability, and Reliability Report of the Integrated Skin Biophysical Measurement Platform: SKENA.
**Figure S1:** (a) Exterior of SKENA including measuring devices and independent computer with analyzing software (b‐f) Comparison of the two series of instruments analyzed by the Pearson correlation coefficient test.
**Table S1:** Coefficient of variation (CV) for repeatability, intra‐class correlation coefficient (ICCs) and standard deviation (SD) for inter‐rater reliability of the SKENA probes taken on healthy skin.

## Data Availability

The data that support the findings of this study are available on request from the corresponding author. The data are not publicly available due to privacy or ethical restrictions.
