# Supplementary material for: Multi‐Parametric Biophysical Analysis for Evaluating Suitability of Energy‐Based Devices and Injectables
Source: J Cosmet Dermatol. 2026 Oct 1;25(10):e71224. doi: 10.1111/jocd.71224 (PMC13631373; doi:10.1111/jocd.71224)
Supplement: Supplementary file 1 [file JOCD-25-e71224-s001.docx]

**Supplementary 1: Technical Validation, Repeatability, and Reliability Report of the Integrated Skin Biophysical Measurement Platform: SKENA**

1. **Regulatory Compliance and Hardware Specifications**

The multi-probe clinical workstation utilized in the primary study (SKENA, V&Co., Seoul, Korea) is a standardized diagnostic system designed for point-of-care biophysical screening. The device is a standardized clinical hardware platform that has officially obtained the Registration of Broadcasting and Communication Equipments from the National Radio Research Agency of Korea under Registration No. R-R-VNCO-VSKINDX001 on February 9, 2026, ensuring full compliance with institutional manufacturing, electrical safety, and operational stability standards. This hardware platform integrates independent sensor probes operating on deeply established skin bioengineering physics, where stratum corneum hydration is evaluated via capacitance metrics, transepidermal water loss is measured via open/closed-chamber vapor density gradient detection, cutaneous color indices for erythema and melanin are quantified through reflectance spectrophotometry, and multi-component mechanical tissue displacement is determined using a vertical suction cup apparatus. These quantitative bioengineering technologies have been shown to capture significant and standardized changes in skin biophysical properties following non-surgical facial rejuvenation treatments [1].

2. **Materials and Methods for Validation**

To establish the concurrent validity, intra-device repeatability, and inter-rater reliability of this integrated system, a preliminary clinical trial was conducted in strict accordance with the ethical principles of the Declaration of Helsinki after obtaining formal approval from the Public Institutional Review Board designated by the Ministry of Health and Welfare of Korea under protocol number P01-202606-01-071. Twenty-six healthy Korean volunteers, comprising 14 females and 12 males with a mean age of 36.5 ± 6.4 years, were enrolled in the trial after providing written informed consent prior to participation. All measurements were taken at a highly standardized anatomical landmark on the cheek, defined precisely by the intersection of the vertical line dropping from the lateral canthus and the horizontal line connecting the nasal ala and tragus, under tightly controlled environmental conditions maintaining an ambient temperature of 22–24°C and a relative humidity of 40–60%. Prior to testing, subjects abstained from topical moisturizing agents for 12 hours and underwent a mandatory 15-minute resting acclimation window to stabilize baseline cutaneous kinetics.

Concurrent validity was assessed using a left-right randomized, non-blinded, contralateral design comparing the SKENA platform directly against globally recognized research standard instruments from Courage & Khazaka (Cologne, Germany), specifically deploying the Corneometer for hydration, the Tewameter for TEWL, and the Cutometer for mechanical elasticity, with pairing concordance analyzed via Pearson correlation coefficients. Intra-device repeatability was determined by a single observer executing five consecutive, parallel measurements at 5-minute intervals on identical skin sites across five subjects, with the resulting data expressed via the Coefficient of Variation (CV%). Inter-rater reliability was measured by two independent observers, blinded to each other's results, who evaluated identical cutaneous zones at 30-minute intervals across five subjects, with data analyzed using a two-way mixed model intra-class correlation coefficient (ICC).

3. **Validation and Reliability Results**

Paired contralateral comparative analyses demonstrated high statistical concordance between the integrated workstation and the standard reference devices, with Pearson correlation coefficients confirming a strong positive correlation for both stratum corneum hydration and transepidermal water loss (r > 0.85, p < 0.05) alongside a statistically significant, moderate correlation for mechanical elasticity (r = 0.669, p < 0.05) (Supplementary figure 1). The platform demonstrated robust intra-device repeatability with single-observer variability remaining exceptionally low for hydration (CV% = 2.42%) and erythema (CV% = 1.34%), while primary elasticity components including total extensibility (U_f_), elastic deformation (U_e_), and total recovery (U_a_) displayed stable boundaries with coefficients bounded between 2.75% and 4.86%. Although viscoelastic creep (U_v_, 7.30%) and immediate elastic recovery (U_r_, 6.19%) exhibited slightly higher variability, the overall repeatability matrix remained highly secure.

Inter-rater reliability analysis revealed excellent operator agreement for hydration (ICC = 0.94) and good agreement for erythema (ICC = 0.88), with composite elasticity metrics displaying acceptable concordance ranging from 0.67 to 0.88, which compares favorably to validation profiles established for other standard diagnostic hardware platforms [2, 3]. Conversely, a lower inter-rater reliability was observed for transepidermal water loss (ICC = 0.43), which underscores the intrinsic physical sensitivity of epidermal vapor tracking to slight probe positioning shifts and highlights the operational necessity for the strict operator standardization protocols deployed throughout the longitudinal assessments of the primary text. The full breakdown of repeatability coefficients and inter-rater reliability metrics across all integrated probes is documented in Supplementary Table 1.
**Technical conclusion**

In summary, this technical verification confirms that the diagnostic hardware yields accurate and repeatable non-invasive skin metrics that align strongly with globally recognized reference instrumentation [2, 3]. When paired with rigorous microclimate stabilization and standardized operator training, the parallel 8-dimensional state vectors generated by this system possess the requisite statistical fidelity to successfully populate the Mahalanobis metric tensor and multi-parametric suitability modeling equations introduced in the primary manuscript. Furthermore, the systematic accumulation of such standardized biophysical datasets across clinical networks establishes a crucial empirical foundation for future AI-driven predictive modeling and personalized cosmetic treatment algorithms [4]. Ultimately, these objective data-driven paradigms are anticipated to facilitate highly customized treatment pathways and improve overall patient satisfaction, marking a significant step toward precision dermatology [5, 6].

 **Figure 1. (a) Exterior of SKENA including measuring devices and independent computer with analyzing software (b-f) Comparison of the two series of instruments analyzed by the Pearson correlation coefficient test.**

| **Instrument** | **Parameters** | **CV%** | **Interobserver** | | |
| --- | --- | --- | --- | --- | --- |
|  |  |  | **Mean** | **SD** | **ICC** |
| **Skin color probe** | Melanin | 0.88 | 33.83 | 0.17 | 0.75 |
|  | Erythema | 1.34 | 25.36 | 0.17 | 0.88 |
| **Hydration probe** | Hydration | 2.42 | 232.2 | 5.7 | 0.94 |
| **TEWL probe** | TEWL | 2.44 | 16.61 | 0.47 | 0.43 |
| **Elasticity probe** | Uf | 2.75 | 2.53 | 0.06 | 0.71 |
|  | Ue | 3.96 | 2.32 | 0.09 | 0.88 |
|  | Uv | 7.30 | 0.20 | 0.03 | 0.67 |
|  | Ur | 6.19 | 0.89 | 0.05 | 0.69 |
|  | Ua | 4.86 | 2.03 | 0.06 | 0.74 |

TEWL, transepidermal water loss

**Table 1. Coefficient of variation (CV) for repeatability, intra-class correlation coefficient (ICCs) and standard deviation (SD) for inter-rater reliability of the SKENA probes taken on healthy skin**

**References**

1. Lee YJ., Kim HT., Lee YJ., et al. Comparison of the effects of polynucleotide and hyaluronic acid fillers on periocular rejuvenation: a randomized, double-blind, split-face trial. J Dermatolog Treat. 2022;33(1):254-260. doi:10.1080/09546634.2020.1748857

2. Hua W, Fan LM, Dai R, et al. Comparison of two series of non-invasive instruments used for the skin physiological properties measurements: the Dermalab from Cortex Technology vs. the series of detectors from Courage & Khazaka. Skin Res Technol. 2017;23:70-78. doi:10.1111/srt.12086

3. Peperkamp K, Verhulst AC, Tielemans HJP, et al., The inter-rater and test-retest reliability of skin thickness and skin elasticity measurements by the Dermalab Combo in healthy participants. Skin Res Technol. 2019;25:787-792 doi:10.1111/srt.12718

4. Thunga S, Khan M, Cho SI, et al. AI in aesthetic/cosmetic dermatology: current and future. J Cosmet Dermatol. 2025;24(1):e16640. doi:10.1111/jocd.16640

5. Kim JH, Choi WS, et al. Water Retention Ability of the Dermis Following Polynucleotide Intradermal Injection Using a Water Distribution Model: Pilot Study. J Cosmet Dermatol. 2025;24(12):e70537. doi: 10.1111/jocd.70537.

6. Kim JH, Choi WS, et al. MRI-Based Assessment of Microfocused Ultrasound-induced Midface Lifting. J Cosmet Dermatol. 2025;24(10):e70477. doi: 10.1111/jocd.70477.
